# Supplementary material for: Two-dimensional metal–organic framework for post-synthetic immobilization of graphene quantum dots for photoluminescent sensing
Source: Commun Chem. 2024 May 11;7:108. doi: 10.1038/s42004-024-01192-5 (PMC11088654; doi:10.1038/s42004-024-01192-5)
Supplement: Supplementary file 2 — Supplementary Information [file 42004_2024_1192_MOESM2_ESM.pdf]

Supplementary information

Two-dimensional metal–organic framework for post-synthetic immobilization of graphene quantum dots for photoluminescent sensing

You-Liang Chen [1], Darwin Kurniawan [2], Meng-Dian Tsai [1], Jhe-Wei Chang [1], Yu-Na Chang [1], Shang-Cheng Yang [1], Wei-Hung Chiang [2,3,4] and Chung-Wei Kung [1]*

[1] Department of Chemical Engineering, National Cheng Kung University, 1 University Road, Tainan City 70101, Taiwan

[2] Department of Chemical Engineering, National Taiwan University of Science and Technology, Taipei City 10607, Taiwan

[3] Sustainable Electrochemical Energy Development (SEED) Center, National Taiwan University of Science and Technology, Taipei City 10607, Taiwan

[4] Advanced Manufacturing Research Center, National Taiwan University of Science and Technology, Taipei City 10607, Taiwan

* Corresponding author

Email: cwkung@mail.ncku.edu.tw

Supplementary Methods

(1) Chemicals

Zirconium (IV) chloride anhydrous ($ZrCl_4$, ACROS Organics, 98%), benzoic acid (BA, Sigma-Aldrich, 99.5%), 1,3,5-tri(4-carboxyphenyl)benzene (H_3BTB, Alfa Aesar, 97%), N,N-dimethylformamide (DMF, ECHO Chemical Co, Ltd., Taiwan, ≥99.8%), dimethyl sulfoxide (DMSO, Duksan Pure Chemicals, 99%), hydrochloric acid (HCl, J. T. Baker, 36.5–38.0%), acetone (ECHO Chemical Co, Ltd., Taiwan, 98%), magnesium chloride hexahydrate ($MgCl_2 \cdot 6H_2O$, Sigma-Aldrich, 98%), zinc chloride ($ZnCl_2$, Sigma-Aldrich, ≥98%), cadmium nitrate tetrahydrate ($Cd(NO_3)_2 \cdot 4H_2O$, Alfa Aesar, ≥98.5%), mercury chloride ($HgCl_2$, ACROS Organics, 99.5%), cobalt chloride anhydrous ($CoCl_2$, Alfa Aesar, 97%), copper chloride dihydrate ($CuCl_2 \cdot 2H_2O$, Sigma-Aldrich, ≥99.0%), nickel nitrate hexahydrate ($Ni(NO_3)_2 \cdot 6H_2O$, Alfa Aesar, 98%), iron(III) sulfate hydrate ($Fe_2(SO_4)_3 \cdot H_2O$, Sigma-Aldrich, 97%), iron(II) acetate anhydrous ($Fe(CO_2CH_3)_2$, Sigma-Aldrich, 95%), chitosan (Sigma-Aldrich, low molecular weight, 50-190 kDa), pyromellitic acid (PMA, Sigma-Aldrich, 96%), 2-phenylphenol (Sigma-Aldrich, 99%), sodium hydroxide (NaOH, Sigma-Aldrich, ≥98%), acetic acid glacial (Fischer Scientific, ≥99.7%), dimethyl sulfoxide-d_6 (DMSO-d_6, Sigma-Aldrich, 99.9 atom% D), sulfuric acid-d_2 solution (D_2SO_4, Sigma-Aldrich, 96–98 wt.% in D_2O, 99.5 atom% D), nitric acid (Honeywell Fluka, ≥65%) and hydrogen peroxide solution (Honeywell Fluka, 30–31%) were used as received. Pt foil (99.95%, 20 mm × 20 mm × 0.1 mm) was obtained from Guv Team International Co., Ltd., Taiwan. Deionized (DI) water was used throughout the whole work.

(2) Synthesis of GQD-1, GQD-2 and GQD-3

GQD-1, GQD-2 and GQD-3 were synthesized by serving pyromellitic acid, chitosan and 2-phenylphenol as the precursors, respectively, with the use of a direct current (DC) argon (Ar) microplasma at ambient conditions. Similar synthesis of GQDs and the detailed experimental setup have been reported in detail in our previous studies.[1-2] For preparing GQD-1, 254 mg of pyromellitic acid was dissolved in 0.5 M NaOH (aq) to prepare an

electrolyte containing 0.1 M of pyromellitic acid. Then, 10 mL of the electrolyte was subjected to a microplasma reaction at a fixed discharge current of 9.6 mA for 60 min to synthesize the solution containing GQD-1. GQD-2 was prepared according to the previous report.[1] Briefly, the chitosan electrolyte was prepared by dissolving 75 mg of chitosan in 10 mL of a 50 mM acetic acid aqueous solution. The obtained electrolyte was then subjected to a microplasma reaction at a fixed discharge current of 8 mA for 20 min to synthesize GQD-2. To synthesize GQD-3, 100 mg of 2-phenylphenol was dissolved in 20 mL of 1.0 M NaOH (aq), followed by the microplasma reaction at a fixed discharge current of 6.5 mA for 90 min.

After the microplasma-based synthesis, both the obtained solutions containing GQD-1 and GQD-3 were neutralized by titrating with 1.0 M HCl (aq), and dialysis against DI water was then performed for 48 h to purify the GQDs. The outer solution was removed and replaced with fresh DI water for every 6 h. For GQD-2, the obtained solution after the microplasma-based synthesis was neutralized by adding 1.0 M NaOH (aq). Then, acetone with a volume ratio of 2:1 to that GQD-2 solution was added to precipitate the unreacted chitosan, followed by the vacuum filtration to remove the precipitate. The obtained solution was further evaporated by using a rotary evaporator equipped with a vacuum controller (Eyela, Japan) at a low pressure of around 10 kPa and a constant temperature of -10 °C maintained by a continuously cooling system. During the evaporation process, the sample vial was partially immersed in a water bath at 25 ºC to remove the acetone. After the removal of acetone, the resulting solution was subjected to dialysis against DI water for 48 h in order to purify GQD-2. The outer solution was removed and replaced with fresh DI water for every 6 h.

After the dialysis, all the three GQDs solutions were subjected to the rotary evaporation until around 0.5 mL of solution remained. Around 3 mL of ethanol was then added to the solution to precipitate the GQDs. GQDs powder was finally obtained by rotary evaporating the solution subsequently followed by drying overnight in a vacuum chamber filled with silica gel. To prepare GQDs solutions with a concentration of 10 mg/mL, 100 mg of each

GQDs powder was re-dispersed in 10 mL of DI water by ultrasonication for 10 min. The obtained GQD solutions were stored in a refrigerator (4-10 °C) for further usage.

(3) Photoluminescence experiments

Steady-state photoluminescence (PL) experiments of all samples were conducted at room temperature by using a FluoroMax® spectrometer (HORIBA Scientific), and the details are described as follows. For collecting the PL spectra of the solid sample, around 10 mg of the MOF-based powder was filled in the holder for the following PL measurements. For collecting the PL spectra of GQD solutions, each GQD solution was diluted by DI water to achieve a concentration of 0.067 mg/mL, and the resulting solution was filled into a cuvette for PL measurements. For the measurements of suspensions containing dispersed GQD-ZrBTB materials, 4.0 mg of MOF-based powder was accurately weighted and homogeneously dispersed in 10 mL of DI water by sonication. After shaking it for 10 s, the suspension was filled into a cuvette for collecting PL spectra. All PL data were collected at a scan rate of 10 nm/s, with the sampling width of 1 nm and integration time of 0.1 s. Excitation and emission slits are both 2 nm for all PL measurements. Cuvette with the size of 1 cm×1 cm×3.5 cm was used for all PL measurements of solutions and suspensions.

Time-resolved PL (TRPL) spectra were recorded by using a HORIBA iHR320 emission mono spectrometer equipped with an amplifier and discriminator module (HORIBA CFD-2G), a laser diode with a peak wavelength of 371 nm (DeltaDiode DD-375L, HORIBA) connected to a diode conditioner (DC-N15-370-10, HORIBA) and a picosecond diode controller (DeltaDiode DD-C1, HORIBA), and a high throughput time-correlated single-photon counting controller (DeltaHub DH-HT, HORIBA). The emission used to record the PL lifetime was adjusted according to the emission peak of each sample under the excitation at 371 nm.

(4) Detection of Cu^{2+} ions and selectivity tests

The following experiments were conducted for the PL detection of Cu^{2+} ions; similar

procedures have been reported in our recently published work.[3] At the beginning, 4.0 mg of GQD-1-ZrBTB was accurately weighted and thereafter dispersed in 5 mL of DI water by sonication. Then, 1.5 mL of the obtained suspension was added into a cuvette containing 1.5 mL of the aqueous solution with a certain concentration of Cu^{2+} ions. After periodically shaking the cuvette for 2 min, the emission spectrum was collected under the excitation at 370 nm. The calibration line was then acquired by recording the emission intensity at 504 nm for the samples containing various concentrations of Cu^{2+} ions, and Stern-Volmer equation (equation (1)) was used to analyze the data.[3] It should be noticed that the final concentration in the 3-mL suspension was defined as the concentration of Cu^{2+} ions in equation (1) and all plots. Stern-Volmer equation is shown as follows,

$$\frac{I_0}{I} = 1 + K_{sv}[\mathrm{Cu}^{2+}] \quad (1)$$

where I_0 is the emission intensity of the sample without adding Cu^{2+} ions, I represents the emission intensity of the suspension with a certain concentration of copper ions ($[Cu^{2+}]$) and K_{sv} represents the Stern–Volmer constant.

For selectivity tests, various salts with diverse metal ions including $MgCl_2$, $ZnCl_2$, $Cd(NO_3)_2$, $HgCl_2$, $CoCl_2$, $Ni(NO_3)_2$, $Fe(III)_2(SO_4)_3$ and $Fe(II)(CO_2CH_3)_2$ were selected to perform the PL measurements. 4.0 mg of GQD-1-ZrBTB was dispersed in 5 mL of DI water by sonication, and 1.5 mL of the obtained suspension was mixed with 1.5 mL of the aqueous solution containing 100 μM of the selected salt. The final concentration of each interferent is thus 50 μM in the sample. Emission spectrum of the sample was then collected after periodically shaking the solution for 2 min.

(5) Instrumentation

Transmission electron microscope (TEM) images of MOF-based materials at low magnifications were collected by using a Hitachi H7500 at an accelerating voltage of 80 kV. High-resolution TEM and high-angle annular dark-field (HAADF) images were then collected by using a JEM-2100F (JEOL) at an accelerating voltage of 200 kV. For pristine

GQDs, TEM measurements were performed by using a field emission gun TEM (FEI Tecnai^{TM} G2 F-20 S-TWIN) at an accelerating voltage of 200 kV. X-ray photoelectron spectroscopy (XPS) measurements for 2D ZrBTB and all MOF-GQD composites were performed on a Theta Probe (Thermo Scientific) equipped with a microfocused electron gun, a multi-position aluminum anode, and a monochromated X-ray source. For pristine GQDs, XPS data were collected by using an ESCALAB Xi^{+} (Thermo Fisher Scientific, United Kingdom) and a PHI 5000 VersaProbe III (ULVAC-PHI. Inc., Japan) equipped with a monochromatic Al Kα X-ray radiation as the source gun. All XPS spectra were corrected by referencing the C 1s peak to 284.8 eV. Raman measurement was performed at room temperature by using a JASCO 5100 spectrometer with a laser excitation wavelength and power of 532 nm and 4 mW, respectively. For preparing Raman samples, the GQD solution was drop-cast on a Si wafer and dried on a hotplate at 50 °C, while the MOF-based solid was spread and compressed on a glass slide. A Si wafer with a Raman shift of 520 cm^{-1} was used to calibrate the spectrometer prior to all measurements. The crystallinity of all materials was investigated by using a powder X-ray diffractometer (PXRD, RIGAKU Ultima IV), with the wavelength of 1.54 Å, voltage of 40 kV, and current of 20 mA. Fourier-transform infrared (FTIR) spectra were collected by using a Nicolet 6700 (Thermo Fisher Scientific). Around 100 mg of KBr and 0.5 mg of the MOF-based material were grinded together, and the obtained mixture was pelletized into a pellet for FTIR measurements. Nitrogen adsorption–desorption isotherms of all solid samples were collected by using an ASAP 2020 (Micromeritics) at 77 K. Every sample was degassed at 110 °C for 4 h before collecting the isotherm. Inductively coupled plasma-optical emission spectrometry (ICP-OES) measurements were performed by using a JY 2000-2 ICP-OES spectrometer (Horiba Scientific). For preparing ICP-OES samples, 4 mg of the MOF-GQD powder was accurately weighted within a microwave vial (2–5 mL, Biotage), and 0.75 mL of sulfuric acid and 0.25 mL of 30% H_2O_2 aqueous solution were added. Thereafter, the vial was crimped and heated at 150 °C in a microwave reactor (Initiator+, Biotage) for 20 min, and the resulting colorless solution was diluted to 40 mL by adding 3 wt% of HNO_3 aqueous solution before ICP-OES

measurements. Similar preparation of ICP-OES samples for Zr-MOFs and their composites has been reported previously.[3-4] Absorption spectra were measured with the use of an UV-2600 (Shimadzu). All the washing and solvent-exchange processes in this work were performed by using a Heraeus™ Megafuge™ 16 centrifuge (Thermo Fisher Scientific) with a TX-400 rotor at the rotating speed of 5000 rpm.

Supplementary Note 1

Raman spectra of GQD-1, GQD-2 and GQD-3 are shown in Figure S1. All the three pristine GQDs exhibit the characteristic peaks located at 1350 cm^{-1} (D band) and 1590 cm^{-1} (G band) in their Raman spectra, which are consistent with the reported Raman spectra of GQDs.[5] The presence of clear Raman peaks also indicates that all quantum dots here are composed of nanometer-sized graphene layers.

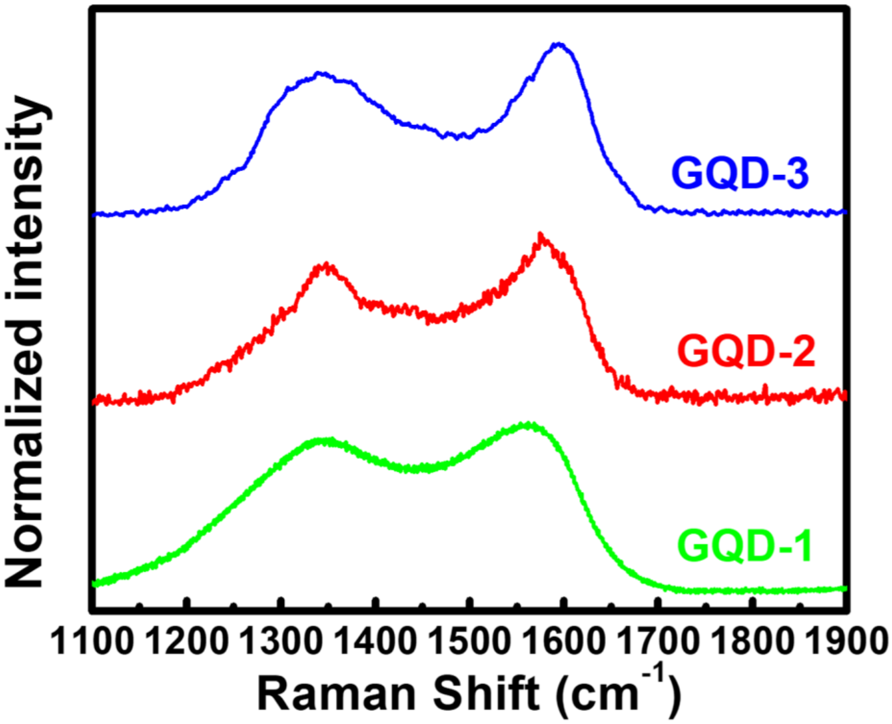

Figure S1. Raman spectra of GQD-1, GQD-2, and GQD-3.

Supplementary Note 2

XPS spectra were further collected to characterize these pristine GQDs. Both GQD-1 and GQD-3 only contain carbon and oxygen, while GQD-2 contains the additional nitrogen owing to its nitrogen-containing precursor, *i.e.*, chitosan. Figure S2 shows the high-resolution XPS spectra of all GQD materials collected in the regions of C 1s, N 1s, and O 1s. To probe the chemical functionalities on the GQDs, spectral deconvolution was conducted on the C 1s,

N 1s and O 1s peaks. The C 1s peaks of GQD-1 and GQD-3 can be deconvoluted into four different peaks comprising of C=C at 284.4 eV,[6] C–O at 285.6-286.0 eV,[7-8] C=O at 287.4 eV and COOH at 288.1 eV (Figure S2(a) and S2(f)).[9] On the other hand, C 1s peaks of GQD-2 can be deconvoluted into five different peaks including C=C (284.4 eV), C–N (285.6 eV), C–O (286.2 eV), C=O (287.4 eV) and COOH (288.1 eV) (Figure S2(c)).[1] O 1s peaks of C–OH (530.9 eV), O=C–OH (532.1 eV) and C–O–C (533.2 eV) can be observed for all GQD materials as well (Figure S2(b), S2(e) and S2(g)).[9] The peak at around 535.5 eV is attributed to the O–Na bond originating from the NaOH added during the synthesis.[1, 9] Figure S2(d) shows the existence of doped nitrogen atoms in GQD-2 with three different configurations including pyridinic N (398.5 eV), amino N (399.3 eV) and pyrrolic N (399.8 eV).[1] The peak at around 397 eV is associated with nitrogen-related species adsorbed on the surface of GQD-2 during the synthetic reaction under atmospheric conditions.[10] Elemental ratios obtained from the XPS data shown in Figure S2 are summarized in Table S1, revealing the significant difference in the chemical functionalities of the three GQDs. All XPS results indicate that the terminal hydroxyl groups and carboxylic groups, which should be both useful for post-synthetically immobilizing GQDs on the 2D Zr-MOF, are present on the surface of all the three GQD materials.

Table S1. Elemental ratios of GQD-1, GQD-2, and GQD-3 calculated from the XPS data.

Materials	Elemental ratios from XPS data	
	N/C	O/C
GQD-1	-	1.46
GQD-2	0.09	0.42
GQD-3	-	0.32

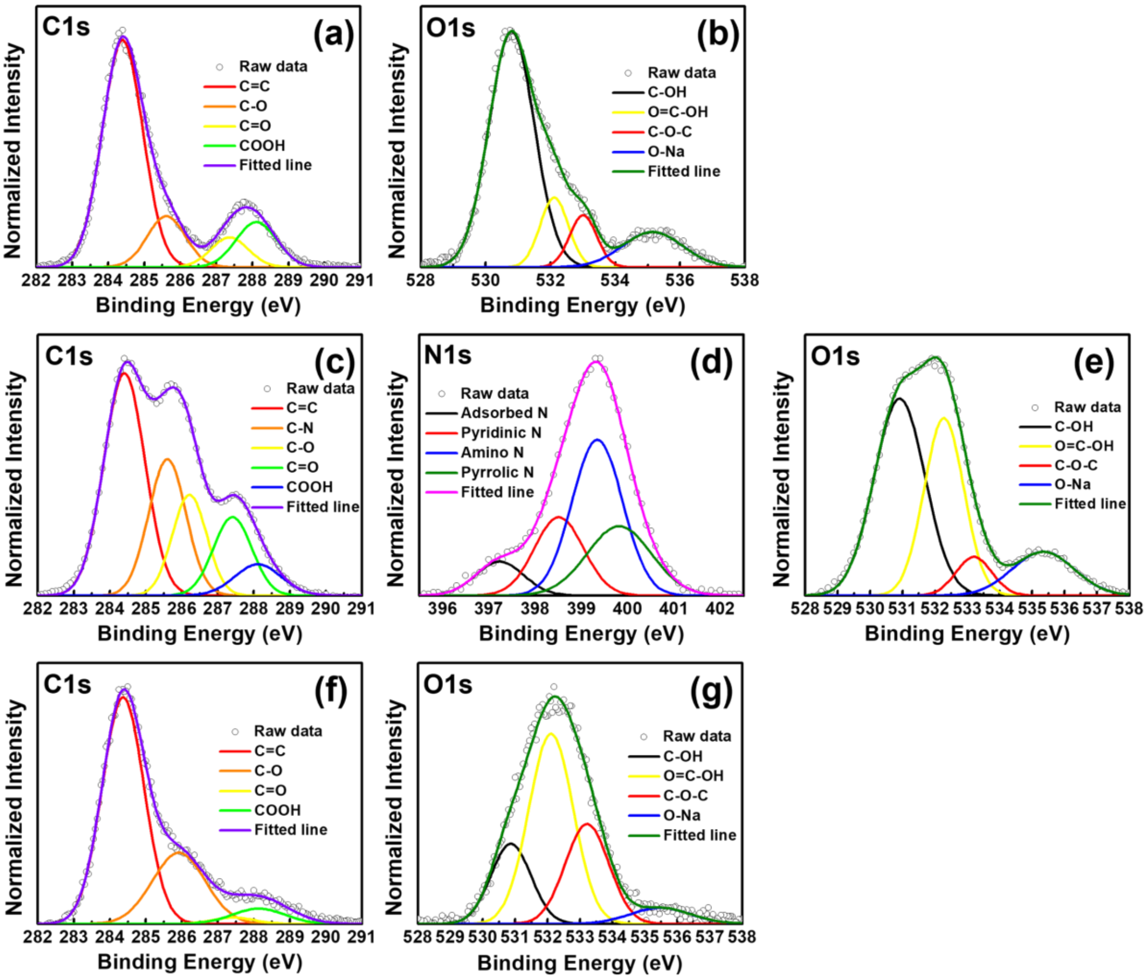

Figure S2. XPS spectra of (a-b) GQD-1 in the regions of C 1s and O 1s, (c-e) GQD-2 in the regions of C 1s, N 1s and O 1s, and (f-g) GQD-3 in the regions of C 1s and O 1s.

Supplementary Note 3

Representative TEM images of GQD-1, GQD-2 and GQD-3 are shown in Figure S3(a), S3(b) and S3(c), respectively. Nanosized spherical particles can be observed in TEM images of all the three samples. To reveal the crystalline structures of GQDs, Fast Fourier Transform (FFT) images were collected. As shown in the bottom left panels of Figure S3(a-c), the unique lattice spacing can be observed in all the three FFT images. High-resolution TEM images were then collected for one particle found in GQD-1, GQD-2 and GQD-3, respectively, and

the data are shown in the bottom right panels of Figure S3(a-c). A lattice spacing of 0.21 nm, which corresponds to the (100) plane of graphene,[1] can be observed, confirming that spherical particles observed in all the three materials are composed of well crystalline graphene quantum dots. Average sizes and size distributions of GQDs were then estimated from the TEM images, and as revealed in Figure S3(d-f), the particle sizes of GQD-1, GQD-2 and GQD-3 are 2.43 ± 0.07, 3.70 ± 0.68 and 4.77 ± 1.00 nm, respectively.

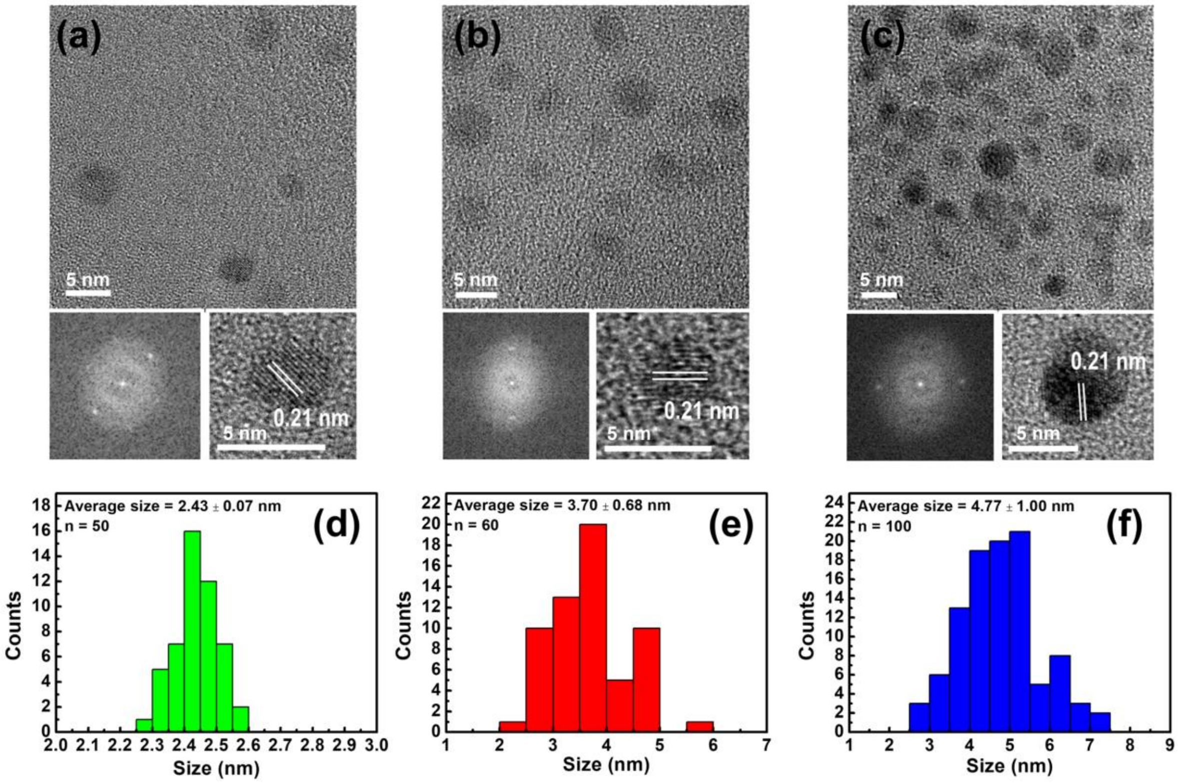

Figure S3. TEM images of (a) GQD-1, (b) GQD-2 and (c) GQD-3. Bottom left panels and bottom right panels of (a-c) show the corresponding FFT images and high-resolution TEM images, respectively. Particle size distributions of (d) GQD-1, (e) GQD-2 and (f) GQD-3.

Supplementary Note 4

Photographs of GQD solutions are shown in Figure S4. It can be seen that all the three GQD solutions exhibit luminescent properties under the illumination of a hand-held UV lamp at 365 nm. However, for all GQD-1, GQD-2 and GQD-3, their dried solid powders do not

reveal obvious luminescence under the illumination at 365 nm (Figure S5); only the GQD-1 powder shows a weak reflection of blue light. This result suggests that the aggregation of GQDs in the form of solid powder can cause the quenching of photoluminescence.

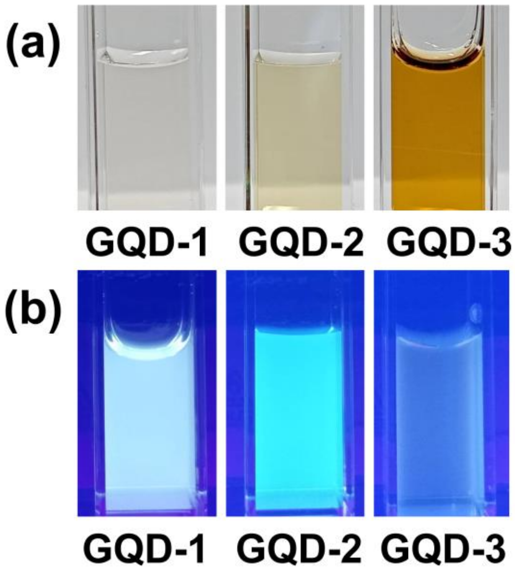

Figure S4. Photographs of aqueous solutions containing (a) GQD-1, GQD-2 and GQD-3 with a concentration of 3 mg/mL, taken under daylight. Corresponding photographs taken under the illumination at 365 nm are shown in (b).

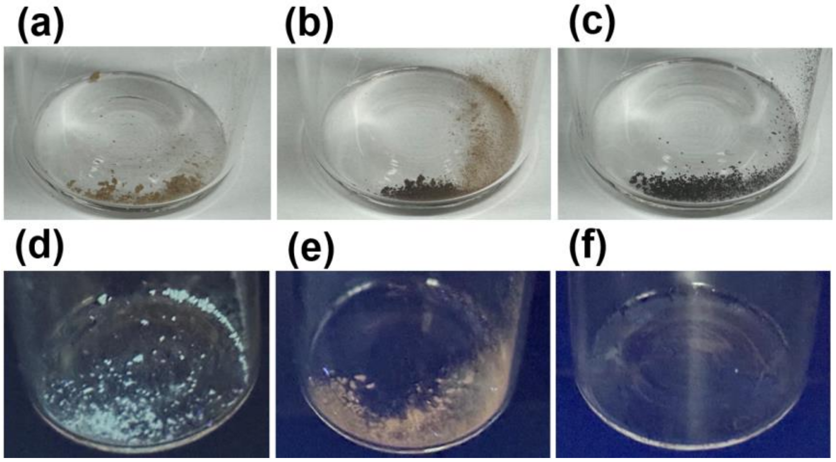

Figure S5. Photographs of dry solid powders of (a) GQD-1, (b) GQD-2 and (c) GQD-3, taken under daylight. Corresponding photographs taken under the illumination at 365 nm are shown in (d), (e) and (f), respectively.

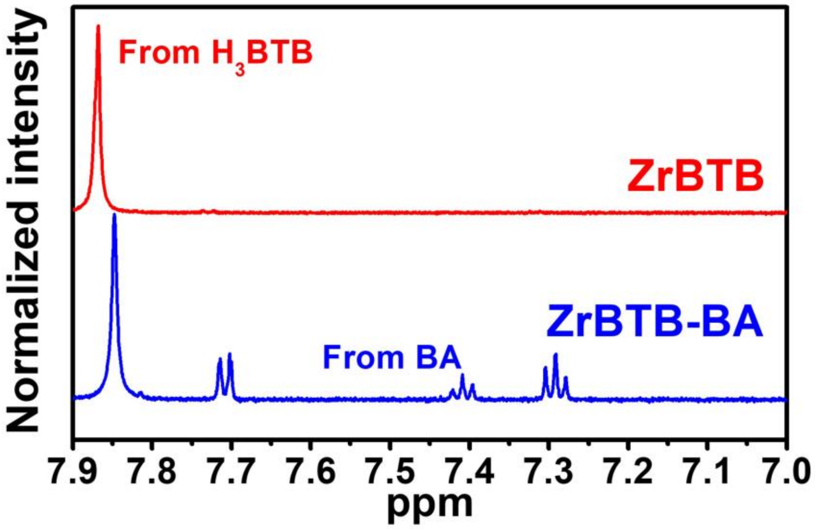

Figure S6. NMR spectra of the digested ZrBTB and digested ZrBTB-BA. Peaks from benzoic acid (BA) are not observable in the spectrum of digested ZrBTB. Detailed synthetic procedures and protocols for NMR measurements can be found in the previous work.[3]

Supplementary Note 5

Tyndall scattering can be well observed after dispersing ZrBTB in all the three GQD solutions by sonication, as revealed in Figure S7.

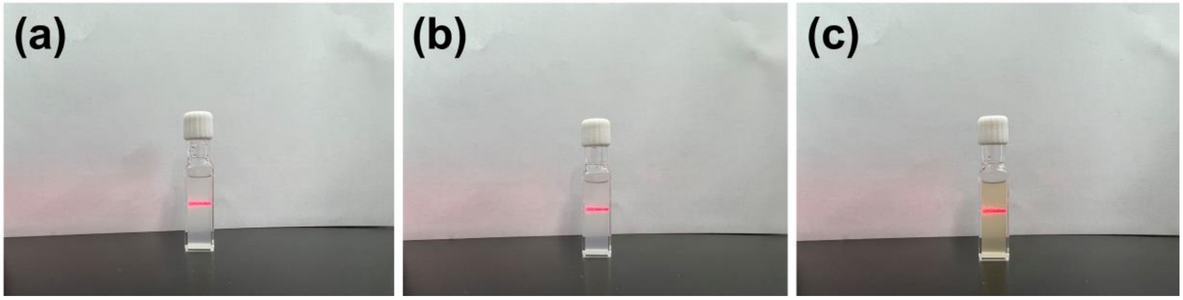

Figure S7. Photos of (a) GQD-1 solution with dispersed ZrBTB, (b) GQD-2 solution with dispersed ZrBTB, and (c) GQD-3 solution with dispersed ZrBTB, with a red laser passing through each suspension from the left side. Concentrations of both ZrBTB and all GQDs are 0.125 mg/mL.

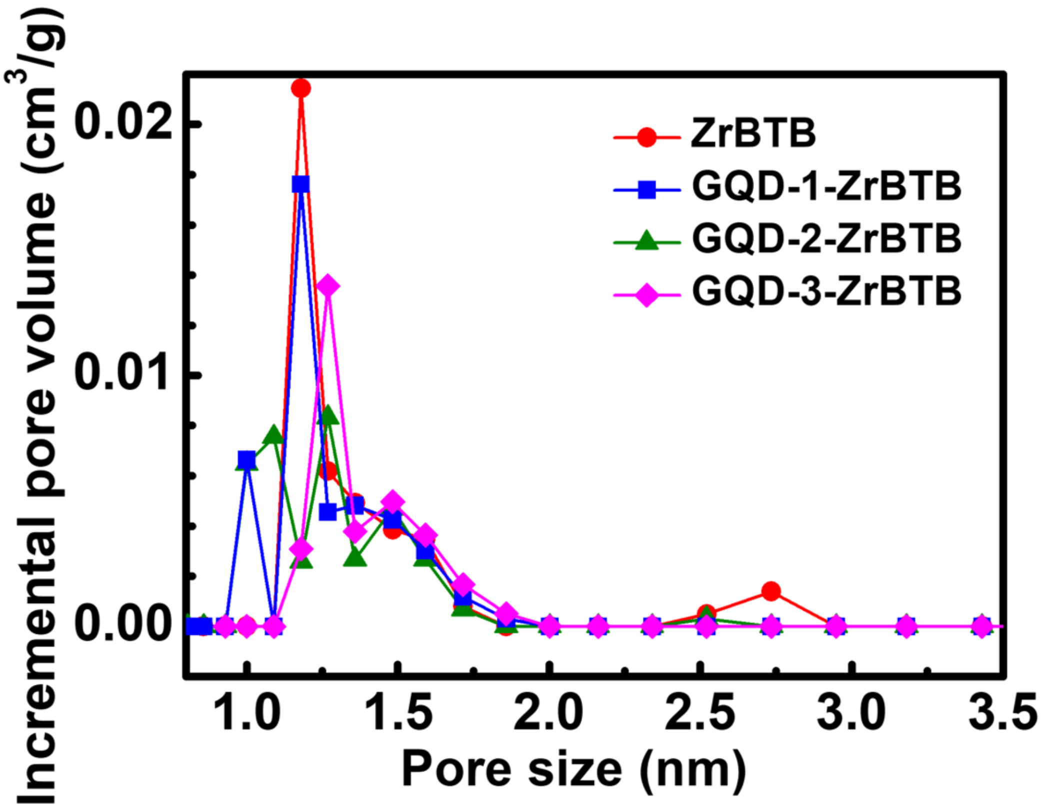

Figure S8. Density functional theory (DFT) pore size distributions of ZrBTB, GQD-1-ZrBTB, GQD-2-ZrBTB and GQD-3-ZrBTB, extracted from the isotherms shown in Figure 2(b) of the main text by using the linear DFT model.

Supplementary Note 6

XPS survey spectra of ZrBTB, GQD-1-ZrBTB, GQD-2-ZrBTB and GQD-3-ZrBTB are shown in Figure S9(a). Signals of C, O and Zr can be found in the spectra of all materials, and the signal of N 1s can be observed in the spectrum of GQD-2-ZrBTB; it confirms the presence of GQD-2 in GQD-2-ZrBTB. XPS spectra of these materials collected in the regions of C 1s and O 1s are also shown in Figure S9(b-c). It should be noticed that the signal of C-N bond from GQD-2, located at around 285.7 eV,[1] can be clearly observed in the C 1s spectrum of GQD-2-ZrBTB (Figure S9(d)), verifying the successful incorporation of GQD-2 in the MOF.

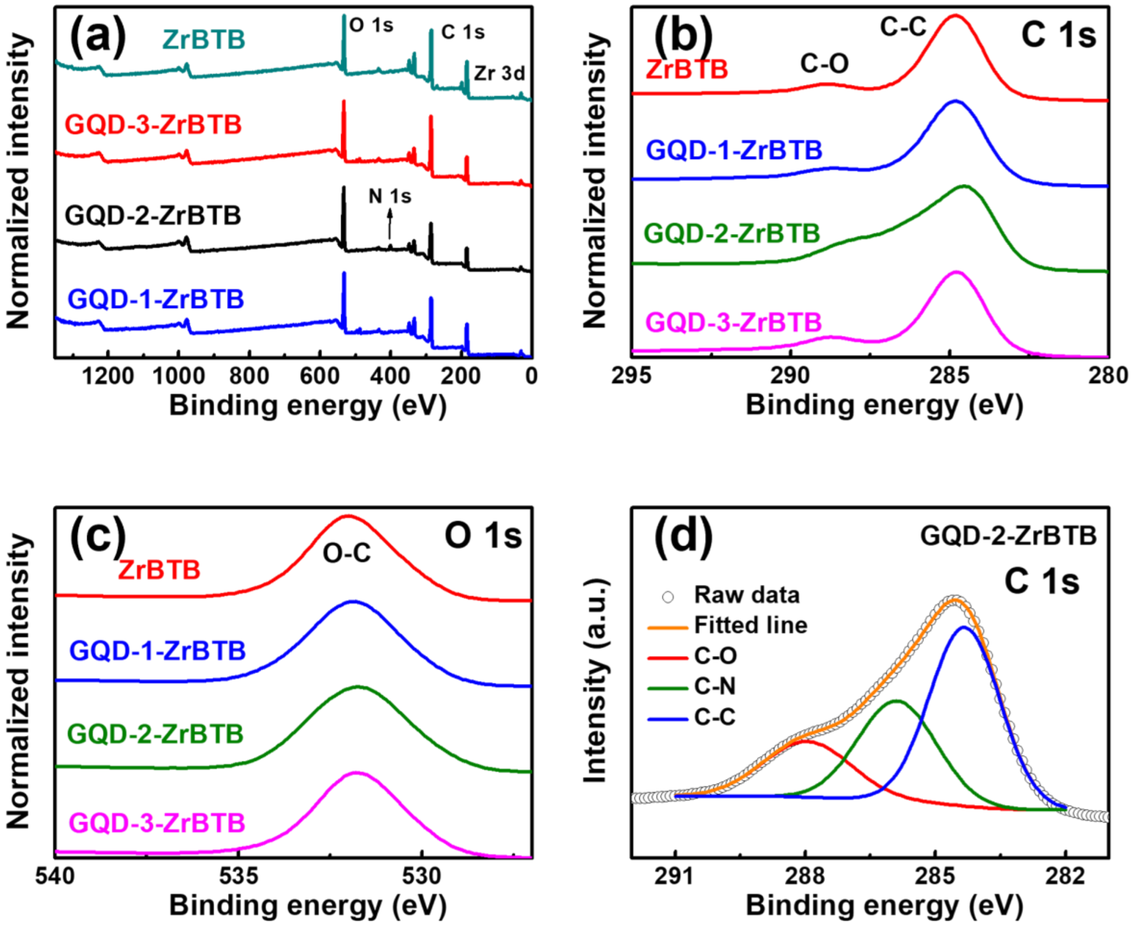

Figure S9. XPS spectra of ZrBTB, GQD-1-ZrBTB, GQD-2-ZrBTB and GQD-3-ZrBTB, collected in the (a) full survey region, (b) C 1s region and (c) O 1s region. (d) XPS spectrum of GQD-2-ZrBTB collected in the C 1s region with peak fitting results.

Supplementary Note 7

FTIR spectra of all MOF-based materials are shown in Figure 2(d) of the main text. Three characteristic peaks of ZrBTB located at 1600, 1540 and 1417 cm^{-1}, which come from the O-C-O and aromatic C=C bonds of ZrBTB,[3, 11] can be observed in FTIR spectra of all the four materials. Another weak and broad peak that is highlighted in yellow in Figure 2(d) can be observed at 1710 cm^{-1}, which corresponds to the uncoordinated carboxylic acid of BTB linkers.[3, 12] This peak indicates that some terminal carboxylic groups should be present, either

at the structural defects in the 2D ZrBTB sheet or on the edge of the sheet. This FTIR peak almost disappears in the spectrum of GQD-2-ZrBTB, implying that during the immobilizing process, a noticeable amount of GQD-2 should be coordinated on these linkers through the terminal -OH groups or amino groups of GQDs. The presence of the peak at 1657 cm^{-1} for the bonds between amino groups of GQD-2 and carboxylate groups from the MOF also supports this conclusion (see Figure 2(d) in the main text). This finding agrees well with the XPS results, suggesting that the majority of GQD-2 are not coordinated on the hexa-zirconium clusters of the MOF.

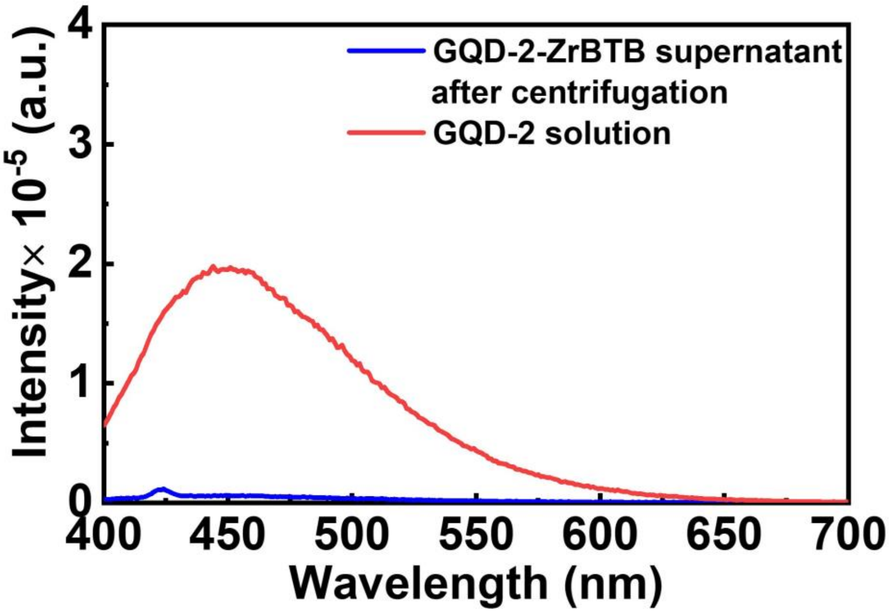

Figure S10. Emission spectra of the GQD-2 solution with a concentration of 0.067 mg/mL and the supernatant obtained by dispersing 4 mg of GQD-2-ZrBTB in 8 mL of water through sonication followed by the centrifugation to remove the MOF solid. Both spectra were collected under the excitation at 370 nm.

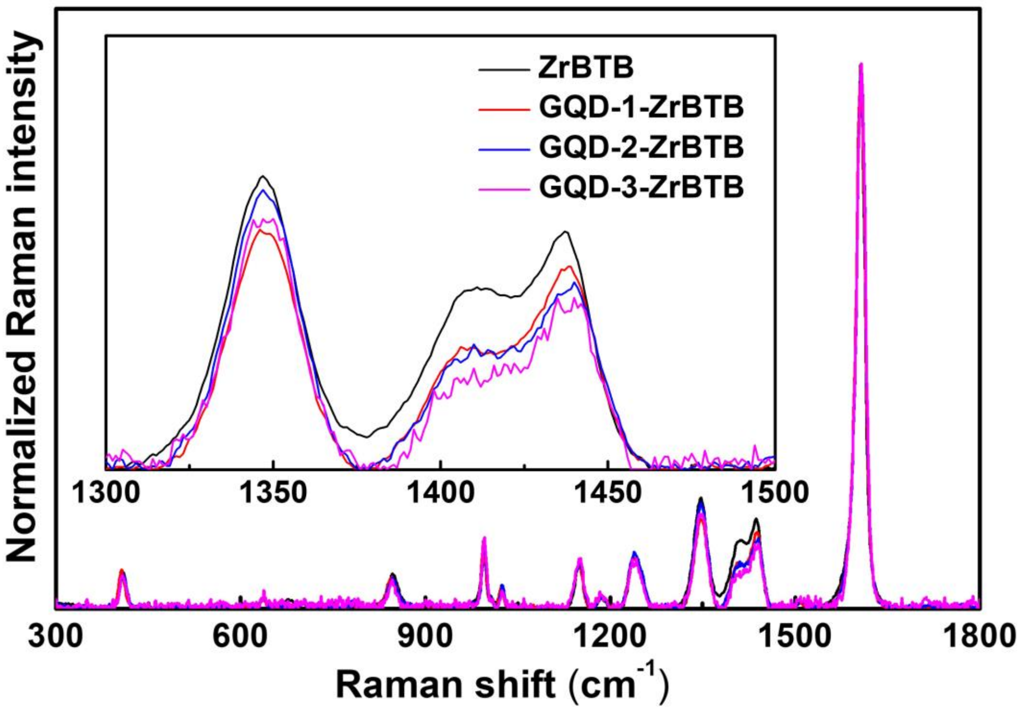

Figure S11. Raman spectra of all MOF-based materials. Inset shows the zoom-in data in the range of the G band and D band of GQDs.

Supplementary Note 8

ICP-OES measurements were conducted for the sample prepared by digesting 4.0 mg of accurately weighted MOF powder followed by diluting the obtained solution to 40 mL; see details in the experimental section. With a higher mass fraction of GQDs present in the composite, the concentration of zirconium in the ICP-OES sample should be lower. This approach has been used to quantify the mass fractions of polymers in various MOF-based composites in our previous studies.[13-15] As a result, the obtained concentrations of zirconium in ICP-OES samples of ZrBTB, GQD-1-ZrBTB, GQD-2-ZrBTB and GQD-3-ZrBTB are 27.2 ppm, 23.7 ppm, 24.4 ppm and 21.7 ppm, respectively. The mass fractions of GQDs in GQD-1-ZrBTB, GQD-2-ZrBTB and GQD-3-ZrBTB were then calculated as 12.8%, 10.3% and 20.2%, respectively.

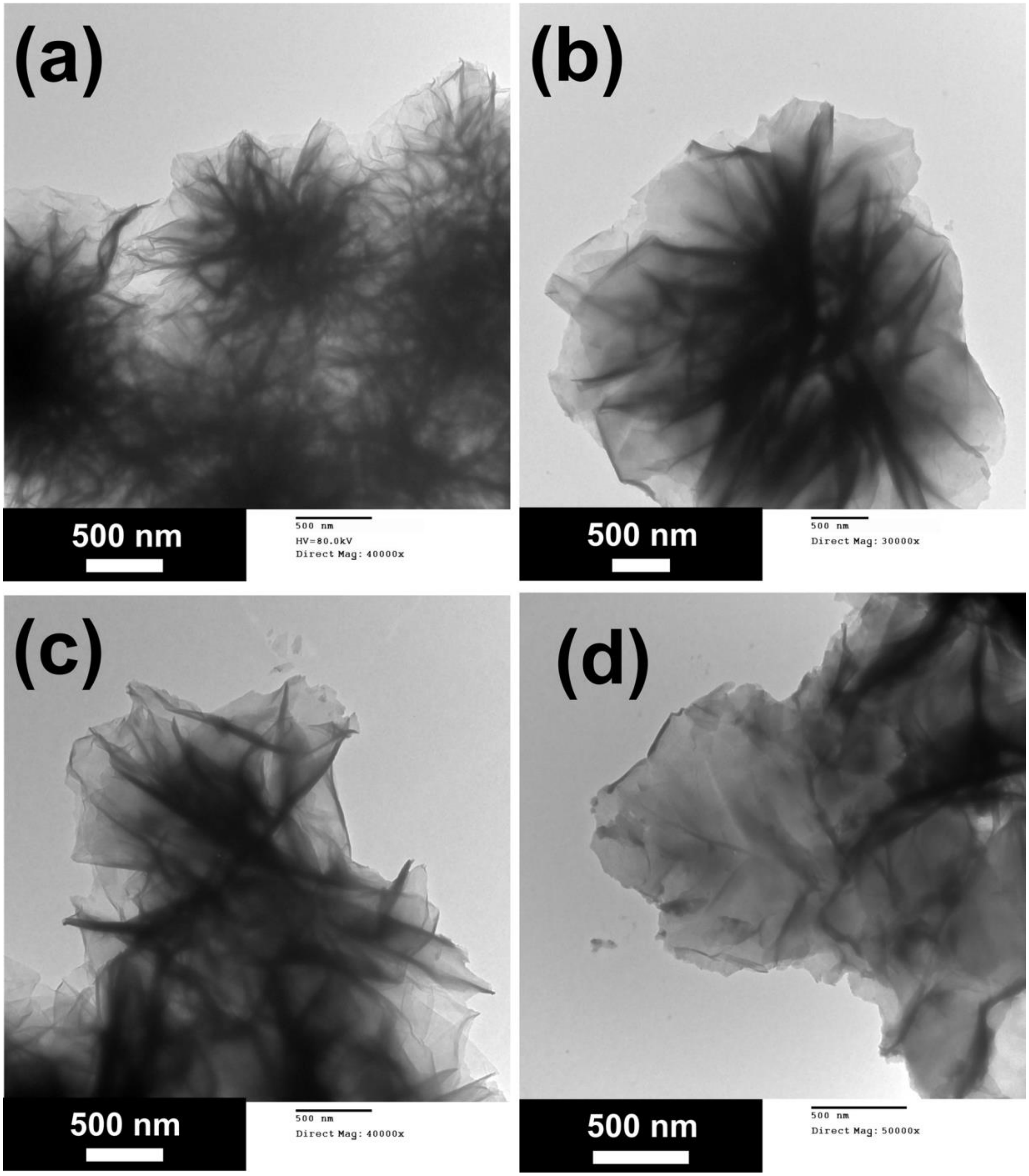

Figure S12. Low-magnification TEM images of (a) ZrBTB, (b) GQD-1-ZrBTB, (c) GQD-2-ZrBTB and (d) GQD-3-ZrBTB.

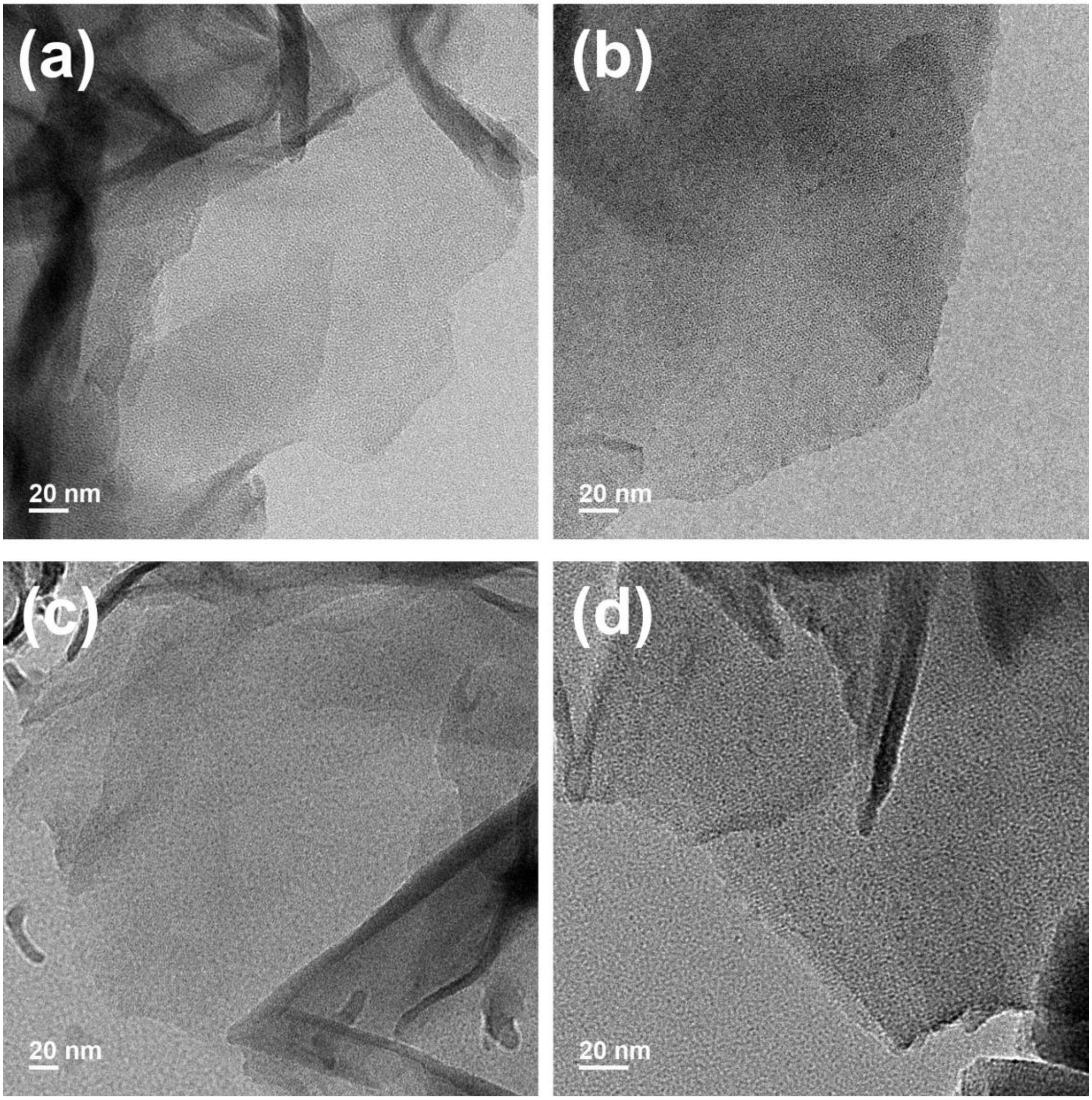

Figure S13. High-magnification TEM images of (a) ZrBTB, (b) GQD-1-ZrBTB, (c) GQD-2-ZrBTB and (d) GQD-3-ZrBTB.

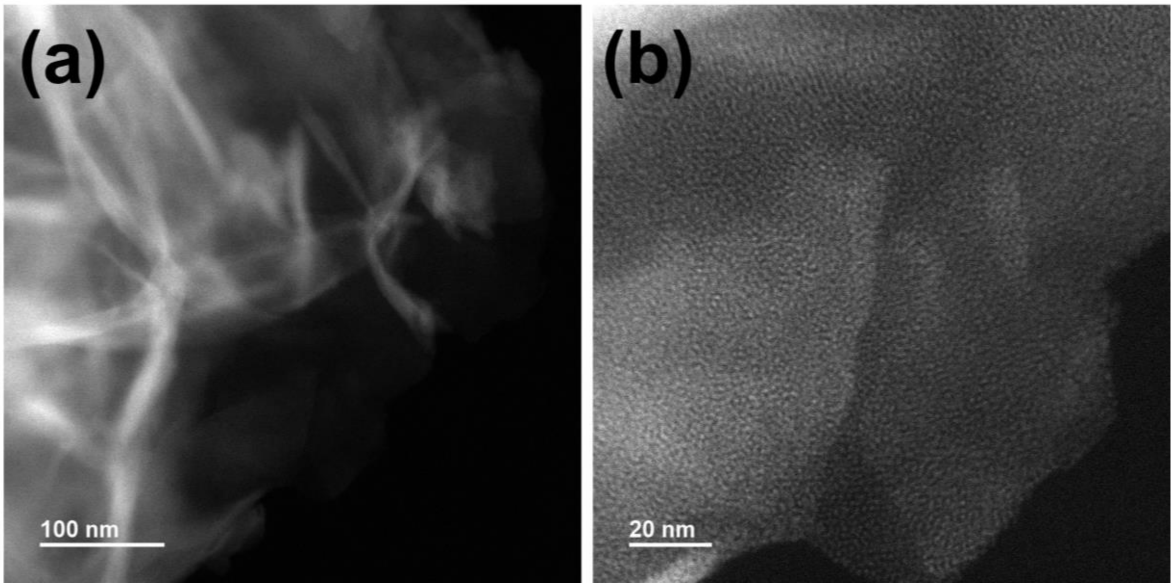

Figure S14. Representative HAADF-STEM images of ZrBTB, collected at a (a) low magnification and (b) high magnification.

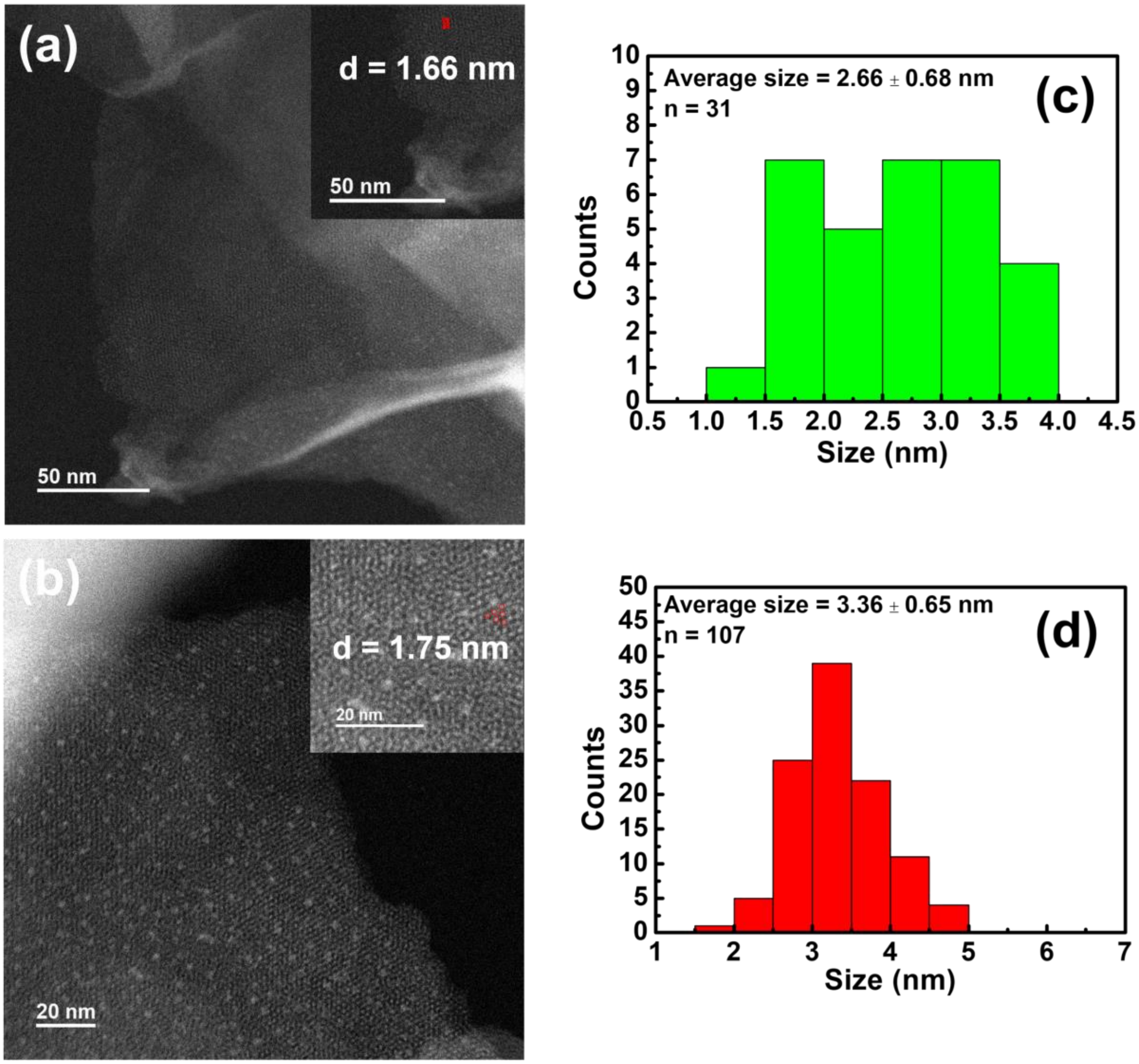

Figure S15. HAADF-STEM images of (a) GQD-1-ZrBTB and (b) GQD-2-ZrBTB. Insets show the images collected at higher magnifications, revealing the lattice fringes of ZrBTB. Particle size distributions of (c) GQD-1-ZrBTB and (d) GQD-2-ZrBTB, estimated from their HAADF-STEM images.

Supplementary Note 9

Figure S16 shows the absorption spectra of the three GQD solutions, revealing a broad absorption band that corresponds to the presence of various electronic states. The absorption bands located at around 250-290 nm and 300-400 nm in each spectrum can be ascribed to the $\pi \rightarrow \pi^*$ transition of C=C bond and $n \rightarrow \pi^*$ transition of functional groups, respectively.[16]

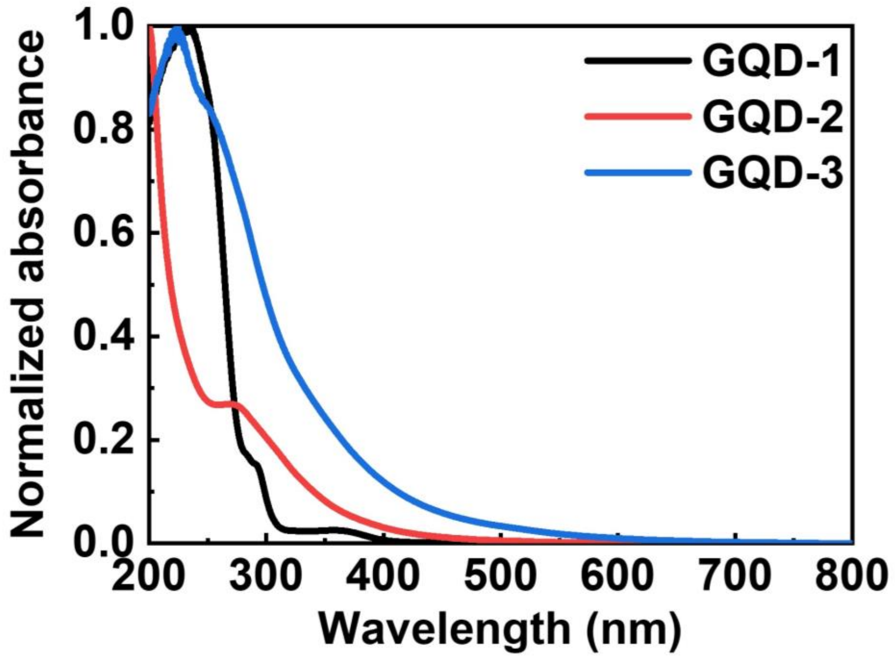

Figure S16. Absorption spectra of aqueous solutions of GQD-1, GQD-2 and GQD-3, with a concentration of 0.067 mg/mL.

Supplementary Note 10

Excitation spectra of all the three GQD solutions were measured by collecting the emission signals near their corresponding wavelengths of maximum emission (see Figure 4(a) of the main text), and the results are shown in Figure S17(a). It can be observed that all the three GQD materials possess the similar optimal wavelengths of excitation at around 350-370 nm, although they reveal quite distinct wavelengths of emission. The excitation spectrum of ZrBTB was also collected. It was already reported that the pristine ZrBTB can show the

emission at around 370 nm under the excitation at 310 nm owing to the luminescent BTB linker.[3] As shown in Figure S17(b), a broad peak centered at around 320 nm can be observed in the excitation spectrum of ZrBTB, and the emission of ZrBTB becomes negligible under the excitation at wavelengths larger than 360 nm. Thus, to maximize the excitation of GQDs as well as avoid the excitation of the ZrBTB itself, all emission spectra of GQDs and MOF-based materials in this study were collected under an excitation at 370 nm.

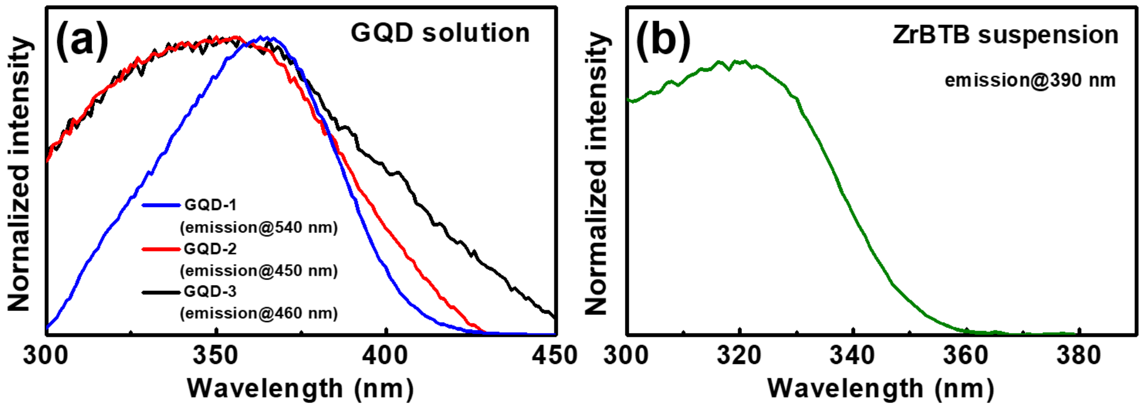

Figure S17. (a) Excitation spectra of aqueous solutions of GQD-1, GQD-2 and GQD-3, with a concentration of 0.067 mg/mL. Emission was collected at 540 nm, 450 nm and 460 nm for GQD-1, GQD-2 and GQD-3, respectively. (b) Excitation spectrum of ZrBTB dispersed in water with a concentration of 0.4 mg/mL. Emission was collected at 390 nm.

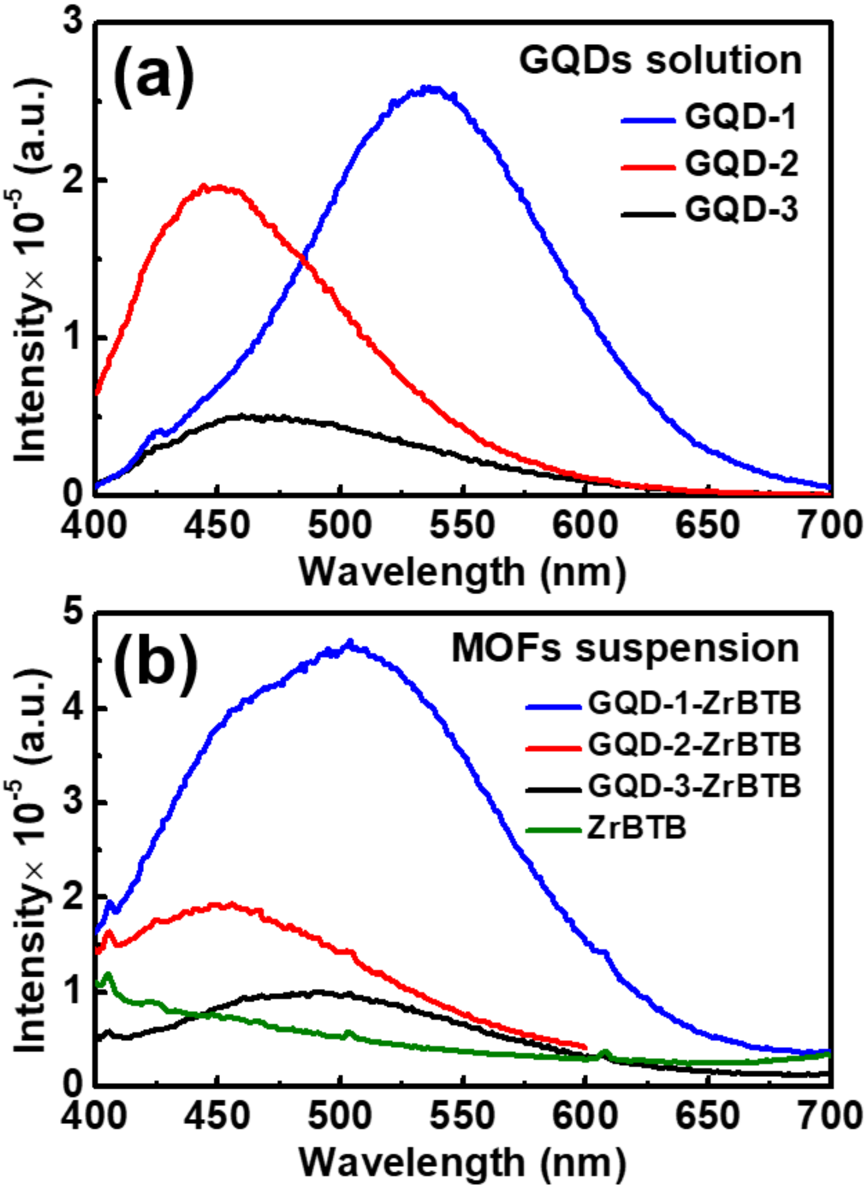

Figure S18. Emission spectra of (a) GQD solutions with a concentration of 0.067 mg/mL and (b) MOF-based materials dispersed in water with a concentration of 0.4 mg/mL. All spectra were collected under the excitation at 370 nm.

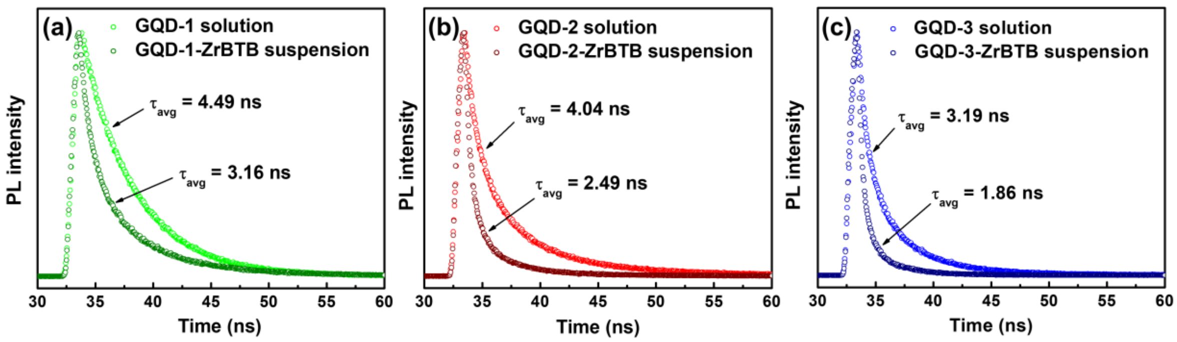

Figure S19. Time-resolved PL data of (a) GQD-1 solution and GQD-1-ZrBTB suspension, (b) GQD-2 solution and GQD-2-ZrBTB suspension, and (c) GQD-3 solution and GQD-3-ZrBTB suspension. The concentration of every GQD solution is 0.067 mg/mL and the concentration of every MOF-based suspension is 0.4 mg/mL. All spectra were collected under the laser excitation at 371 nm. Calculated values of average PL lifetime (τ_{avg}) are listed in each plot.

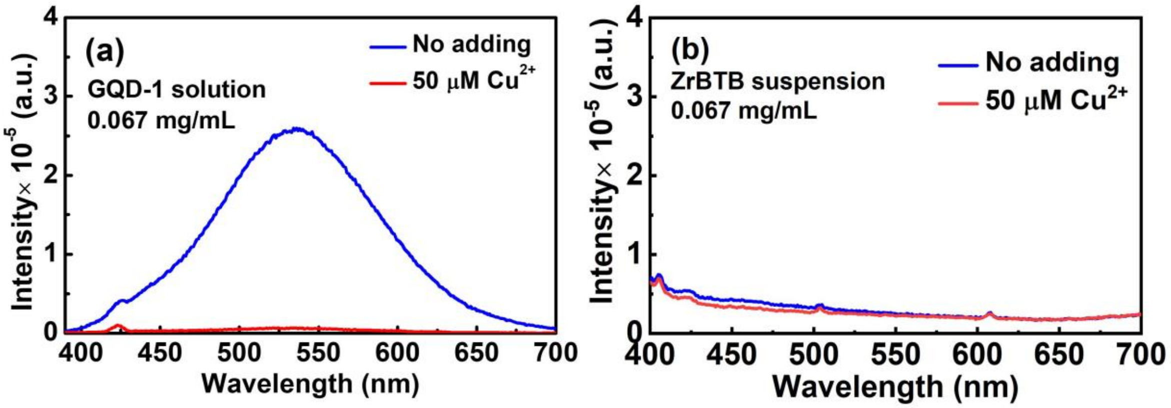

Figure S20. Emission spectra of (a) GQD-1 solutions before and after adding 50 μM of Cu^{2+} ions and (b) ZrBTB suspensions before and after adding 50 μM of Cu^{2+} ions, measured under an excitation at 370 nm. Concentrations of both GQD-1 and ZrBTB are 0.067 mg/mL.

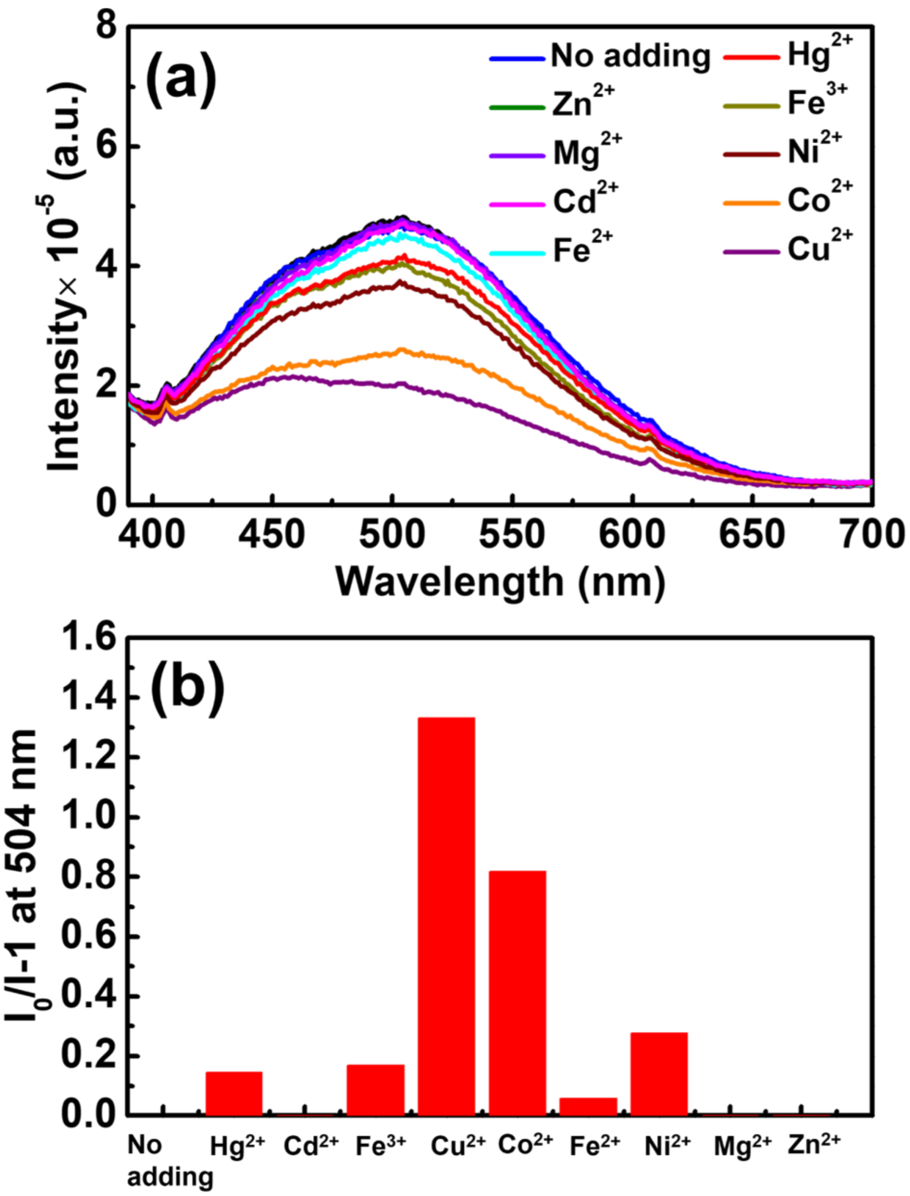

Figure S21. (a) Emission spectra of GQD-1-ZrBTB dispersed in water containing various metal ions, measured under an excitation at 370 nm. Concentration of all metal ions is 50 μM, and the concentration of GQD-1-ZrBTB is 0.4 mg/mL. (b) Intensities at 504 nm extracted from (a).

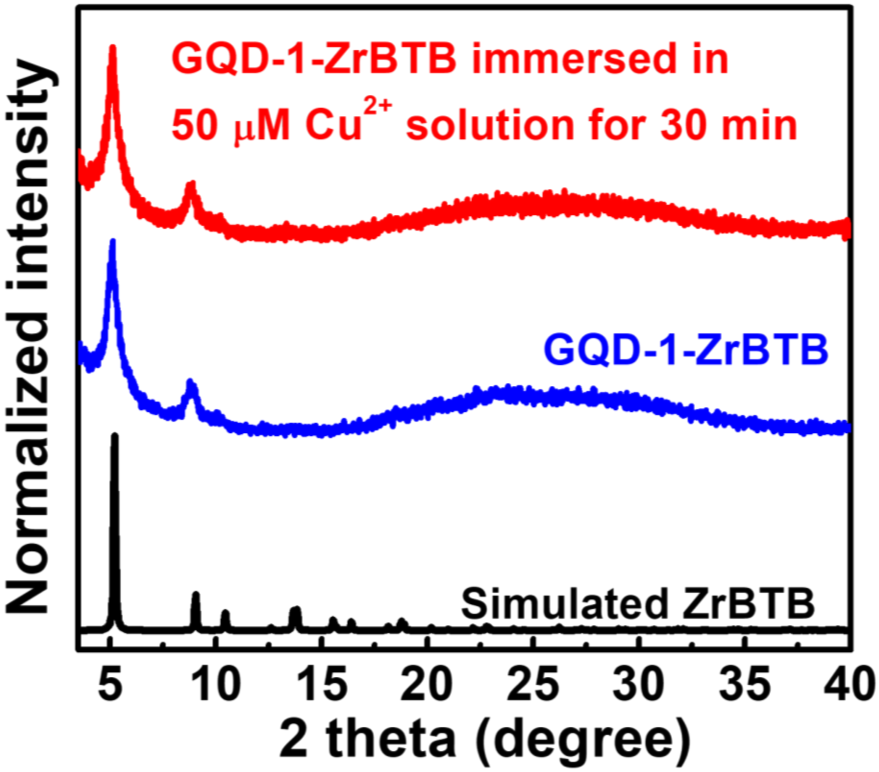

Figure S22. PXRD patterns of the fresh GQD-1-ZrBTB and GQD-1-ZrBTB after the exposure to an aqueous solution containing 50 μM of Cu^{2+} for 30 min. The material after the exposure was washed with water for three times followed by the solvent exchange with acetone and activation before the PXRD measurement. Simulated pattern of ZrBTB is also shown for comparison.

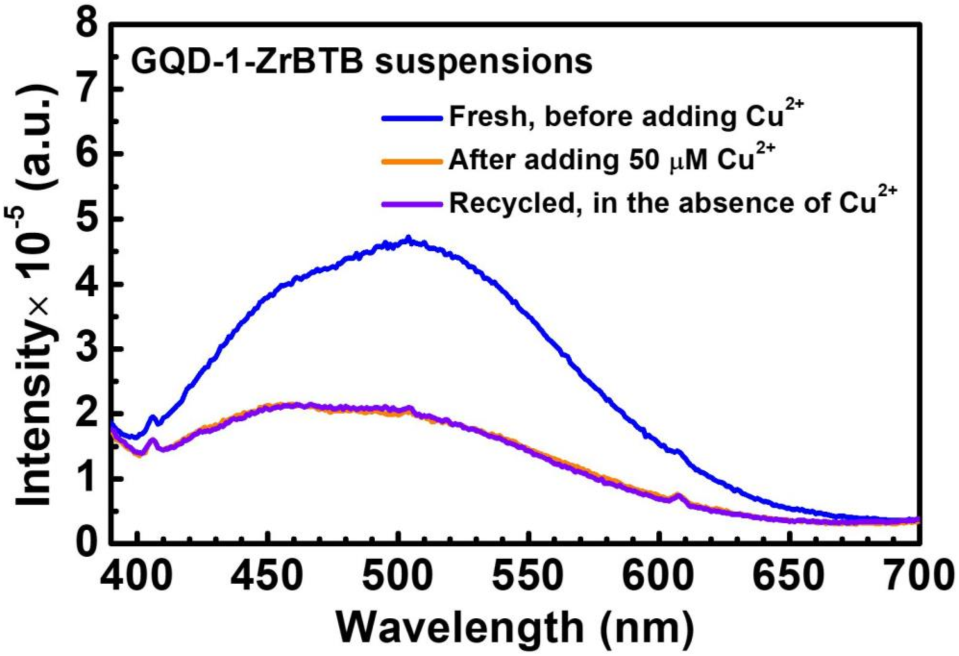

Figure S23. Emission spectra of the fresh GQD-1-ZrBTB dispersed in water in the absence and in the presence of 50 μM of Cu^{2+} ions and the aqueous suspension of the recycled GQD-1-ZrBTB after the exposure to Cu^{2+} ions. Concentrations of all suspensions are 0.4 mg/mL, and all spectra were collected under an excitation at 370 nm. The recycled GQD-1-ZrBTB is the same sample for the PXRD measurement shown in Figure S22.

Supplementary References

1. Kurniawan, D., Rahardja, M. R., Fedotov, P. V., Obraztsova, E. D., Ostrikov, K. K. & Chiang, W.-H. Plasma-bioresource-derived multifunctional porous NGQD/AuNP nanocomposites for water monitoring and purification. *Chem. Eng. J.* **451,** 139083 (2023).

2. Kurniawan, D. & Chiang, W.-H. Microplasma-enabled colloidal nitrogen-doped graphene quantum dots for broad-range fluorescent pH sensors. *Carbon* **167,** 675-684 (2020).

3. Chen, Y.-L., Shen, C.-H., Huang, C.-W. & Kung, C.-W. Terbium-modified two-dimensional zirconium-based metal–organic frameworks for photoluminescent detection of nitrite. *Mol. Syst. Des. Eng.* **8,** 330-340 (2023).

4. Mondloch, J. E., Bury, W., Fairen-Jimenez, D., Kwon, S., DeMarco, E. J., Weston, M. H., Sarjeant, A. A., Nguyen, S. T., Stair, P. C., Snurr, R. Q., Farha, O. K. & Hupp, J. T. Vapor-phase metalation by atomic layer deposition in a metal–organic framework. *J. Am. Chem. Soc.* **135,** 10294-10297 (2013).

5. Liu, F., Jang, M. H., Ha, H. D., Kim, J. H., Cho, Y. H. & Seo, T. S. Facile synthetic method for pristine graphene quantum dots and graphene oxide quantum dots: Origin of blue and green luminescence. *Adv. Mater.* **25,** 3657-3662 (2013).

6. Ye, X., Xiang, Y., Wang, Q., Li, Z. & Liu, Z. A red emissive two-photon fluorescence probe based on carbon dots for intracellular pH detection. *Small* e1901673 (2019).

7. Zhang, S., Zhu, J., Qing, Y., Wang, L., Zhao, J., Li, J., Tian, W., Jia, D. & Fan, Z. Ultramicroporous carbons puzzled by graphene quantum dots: Integrated high gravimetric, volumetric, and areal capacitances for supercapacitors. *Adv. Funct. Mater.* **28,** 1805898 (2018).

8. Wei, S., Yin, X., Li, H., Du, X., Zhang, L., Yang, Q. & Yang, R. Multi-color fluorescent carbon dots: Graphitized sp^2 conjugated domains and surface state energy level Co-modulate band gap rather than size effects. *Chem. Eur. J.* **26,** 8129-8136 (2020).

9. Kurniawan, D., Sharma, N., Rahardja, M. R., Cheng, Y.-Y., Chen, Y.-T., Wu, G.-X., Yeh, Y.-Y., Yeh, P.-C., Ostrikov, K. K. & Chiang, W.-H. Plasma nanoengineering of bioresource-derived graphene quantum dots as ultrasensitive environmental nanoprobes. *ACS Appl. Mater.*

Interfaces **14,** 52289−52300 (2022).

10. Baldwin, E. K. & Friend, C. M. Summary abstract: X-ray photoelectron spectroscopy studies of NO and N_2O on clean and oxygen covered W(100) surfaces. *J. Vac. Sci. Technol. A* **4,** 1407-1407 (1986).

11. Li, Y. & Yang, R. T. Gas adsorption and storage in metal−organic framework MOF-177. *Langmuir* **23,** 12937-12944 (2007).

12. Chen, P., Liu, Y., Hu, X., Liu, X., You, E.-M., Qian, X., Chen, J., Xiao, L., Cao, L., Peng, X., Zeng, Z., Jiang, Y., Ding, S.-Y., Liao, H., Wang, Z., Zhou, D. & Wang, C. Probing surface structure on two-dimensional metal-organic layers to understand suppressed interlayer packing. *Nano Res.* **13,** 3151-3156 (2020).

13. Song, Y.-D., Ho, W. H., Chen, Y.-C., Li, J.-H., Wang, Y.-S., Gu, Y.-J., Chuang, C.-H. & Kung, C.-W. Selective formation of polyaniline confined in the nanopores of a metal–organic framework for supercapacitors. *Chem. Eur. J.* **27,** 3560-3567 (2021).

14. Tsai, M.-D., Chen, Y.-L., Chang, J.-W., Yang, S.-C. & Kung, C.-W. Sulfonate-functionalized two-dimensional metal–organic framework as a “dispersant” for polyaniline to boost its electrochemical capacitive performance. *ACS Appl. Energy Mater.* **6,** 11268-11277 (2023).

15. Tsai, M.-D., Wang, Y.-C., Chen, Y.-L., Chen, Y.-H., Shen, C.-H. & Kung, C.-W. Selectively confined poly(3,4-ethylenedioxythiophene) in the nanopores of a metal–organic framework for electrochemical nitrite detection with reduced limit of detection. *ACS Appl. Nano Mater.* **5,** 12980-12990 (2022).

16. Liu, M. Optical properties of carbon dots: A review. *Nanoarchitectonics* **1,** 1-12 (2020).
